# Pre-operative diagnosis and successful surgery of a strangulated internal hernia through a defect in the falciform ligament: a case report

**DOI:** 10.1186/1752-1947-6-206

**Published:** 2012-07-18

**Authors:** Hironori Shiozaki, Shintaro Sakurai, Kazuki Sudo, Gen Shimada, Hiroshi Inoue, Seiji Ohigashi, Gautam A Deshpande, Osamu Takahashi, Hisashi Onodera

**Affiliations:** 1 Department of GI Surgery, St Luke’s International Hospital, 9-1 Akashi-cho, Chuo-ku, Tokyo, 104-0044, Japan; 2 Division of General Internal Medicine, Department of Medicine, St. Luke’s International Hospital, 9-1 Akashi-cho, Chuo-ku, Tokyo, 104-0044, Japan; 3 University of Hawaii, Department of Internal Medicine, Honolulu, HI, USA

## Abstract

**Introduction:**

Internal hernia within the falciform ligament is exceedingly rare. A literature search revealed only 14 cases of internal herniation of the small bowel through a congenital defect of the falciform ligament, most of which were found intra-operatively.

**Case presentation:**

A 77-year-old Japanese woman presented to our emergency department with sudden hematemesis, occurring at least four to five times over a 12-hour period. No ulcer or gastrointestinal bleeding was detected on gastroendoscopy. A 40mm mass in the inferior lobe of the right lung was found on a chest X-ray, and our patient’s symptoms were therefore initially ascribed to aspirated blood from lung tumor-associated hemoptysis. However, our patient continued to show signs of severe abdominal pain and decreased urine output despite aggressive hydration, leading her examining physicians to search for a possibly severe, occult abdominal pathology. On emergent computed tomography imaging, we found an acute strangulated internal hernia within the falciform ligament. Diagnosis was made by helical computed tomography, permitting rapid surgical intervention.

**Conclusions:**

Our findings on computed tomography imaging assisted with the pre-operative diagnosis and enabled us to make a rapid surgical intervention. Early diagnosis may help preclude significant strangulation with unnecessary resection.

## Introduction

While a possibility, internal hernia is not a common etiology for small bowel obstruction. Among the differential diagnoses for internal hernia, herniation through a defect in the falciform ligament is one of the most rare findings. A literature search revealed only 14 cases of internal herniation of the small bowel through a congenital defect of the falciform ligament, most of which were found intra-operatively. We report a case of pre-operative diagnosis of herniation through the falciform ligament made by characteristic computed tomography (CT) findings.

## Case presentation

A 77-year-old Japanese woman presented to our emergency department with hematemesis. As given on the report from her nursing home, she had experienced a sudden onset of vomiting dark red fluid at least four or five times over the prior 12 hours. Her medical history included hypertension for six years, and cerebral hemorrhage three years previously with left-sided paresis and decreased mentation. Her medications included a calcium-channel blocker, an atypical anti-psychotic (sulpiride), and sodium valproate.

On physical examination, she was afebrile, had a pulse rate of 117 beats per minute, and a blood pressure of 111/62mmHg. The differential diagnosis included ulcer, gastrointestinal (GI) tumor, angiodysplasia and diverticulitis of the small intestine, enteroaortic fistula associated with dissection or aneurysm, and small bowel ischemia with necrosis. However, no GI bleeding or evidence of ulcer was detected on emergent gastroendoscopy. A primary pulmonary source was also considered, and a chest X-ray revealed a 40mm mass in the inferior lobe of the right lung; our patient’s symptoms were initially ascribed to swallowed and regurgitated blood from lung tumor-associated hemoptysis. However, subsequent serial physical examinations of the abdomen revealed signs of continuing, severe abdominal pain, with our patient grimacing during the examination, though without significant rigidity or guarding. Full characterization of the pain was limited by our patient’s low level of consciousness due to past cerebrovascular accident. In addition to abdominal pain, our patient continued to have decreased urine output despite aggressive hydration, leading us to search for a possibly severe, occult abdominal pathology.

**Figure 1:**
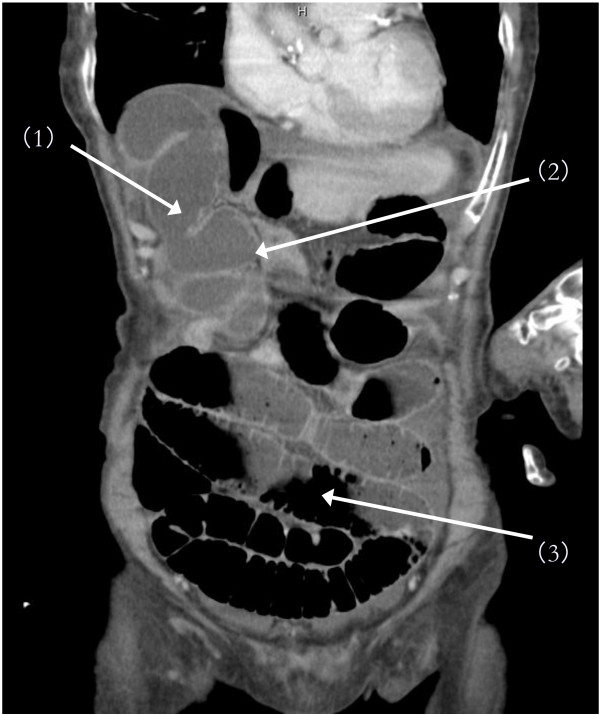
**Pre-operative abdominal helical computed tomography (CT): coronal view.** A membranous structure (1) was visualized in the center of the upper abdomen between the diaphragm and the left lobe of the liver, and dilated, edematous intestine (2) was seen on the right side of it, while air-dilated intestine (3) was seen on the left side and in lower abdomen.

**Figure 2:**
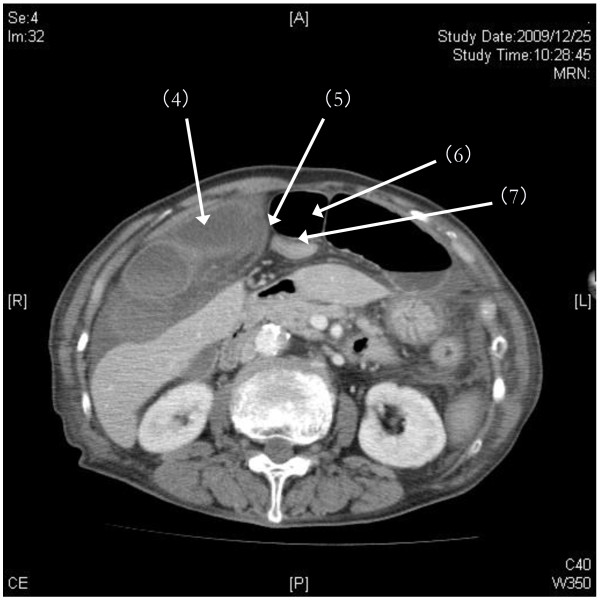
**Pre-operative abdominal helical computed tomography (CT): horizontal view 1**. On horizontal views, a closed loop of intestine (4) was identified against the liver (S4 and S5); the membranous structure (5) was identified as the falciform ligament in the center of the abdomen. On the left side, dilation of proximal intestines (6) and collapse of distal intestine (7) was seen.

**Figure 3:**
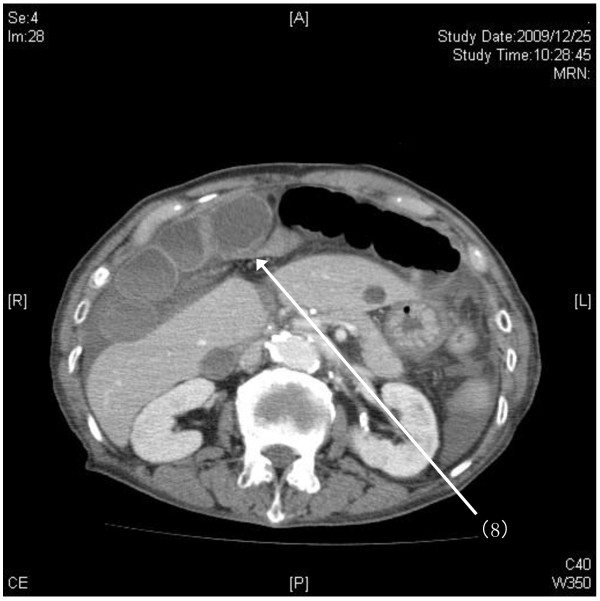
**Pre-operative abdominal helical computed tomography (CT): horizontal view 2.** Another cephalic horizontal view showed the strangulated distal intestine through a hilar defect (8) in the falciform ligament.

Laboratory test results revealed a white blood cell count of 18,000 cells/μL, C-reactive protein level of 9.03mg/dL, lactate dehydrogenase level of 342IU/L, and creatine kinase level of 1324IU/L. With an increasing likelihood of abdominal pathology, emergent CT imaging was ordered. Abdominal helical CT revealed ascites and dilated small bowel. On coronal views, a membranous structure was visualized in the center of the upper abdomen between the diaphragm and the left lobe of the liver; dilated, edematous intestine was seen on the right side of it, while air-dilated intestine was seen on the left side and in the lower abdomen (Figure 1). We suspected that air introduced by prior gastroendoscopy may have been the cause of this dilation. On horizontal views, a closed loop of intestine was identified against the liver (S4 and S5); the membranous structure was identified as the falciform ligament in the center of the abdomen. On the left side, dilation of proximal intestines and collapse of distal intestine was seen (Figure 2). Another cephalic horizontal view showed strangulated distal intestine through a hilar defect in the falciform ligament (Figure 3). Based on the radiographic assessment, a pre-operative diagnosis of strangulated internal hernia through a defect in the falciform ligament was made.

In addition to ascites, CT demonstrated strangulated intestine with decreased contrast enhancement. We suspected necrosis of the strangulated intestines through the falciform ligament defect. Our patient was therefore taken to the operating room urgently. Abdominal exploration revealed bloody ascites and a gangrenous bowel. Approximately 50cm of small intestine, 2m distal to Treitz’ ligament, was looped over a defect in the falciform ligament. Prolonged strangulation had resulted in massive necrosis of tissue. The round ligament of the liver was cut, opening the falciform ligament and releasing the herniated bowel loop. The necrotic bowel was resected and primary anastomosis was performed. Our patient had a stable post-operative course with no gastrointestinal complications and was subsequently transferred five days after surgery for investigation of lung cancer.

## Discussion

The incidence of internal hernias is 0.2% to 2%, and only a proportion of that number cause small bowel obstruction [1]. The types of internal hernias and their relative incidences are as follows: paraduodenal, 53%; pericecal, 13%; foramen of Winslow, 8%; transmesenteric, 8%; intersigmoid, 6%; supravesicular and pelvic, 6%; transmental, 1% to 4% [2,3]. The incidence of internal hernia through a defect in the falciform ligament is estimated at only 0.2%, with an even lower figure caused by congenital anomaly [4]. A thorough literature search from 1948 to 2010 found only 14 case reports of small bowel obstruction caused by internal hernia through a congenital defect in the falciform ligament [5,6].

The etiology of defects in the falciform ligament is diverse, and includes congenital anomalies, trauma, inflammation, and iatrogenic causes. Some reports in the literature note these internal hernias occur after laparoscopic surgery, with defects in the falciform ligament introduced during insertion of the port cannula during laparoscopic fundoplication or cholecystectomy [7,8].

Congenital defects in the falciform ligament have also been reported. Development of the foregut begins after the respiratory diverticulum, and extends to and includes the hepatocystic diverticulum. It appears at the distal end of the foregut at four weeks of the intra-uterine period. The falciform ligament subsequently develops from the septum between the liver and anterior abdominal wall. Hypoplasia of the falciform ligament gives rise to the noted defect during this process [9].

Internal hernias are more often reported in early childhood or late pregnancy. In late pregnancy, the gravid uterus expands, pressing the small bowel into to the upper abdomen, allowing relatively easy passage through congenital falciform ligament defects [10]. In our patient’s case, however, there was no surgical history and our patient was an older person. Close intra-operative exploration did not reveal an acquired cause of internal hernia through the falciform ligament; we therefore assume this to be a case of congenital defect causing spontaneous internal hernia in an older patient.

Abdominal helical CT imaging demonstrated several specific findings. First, marked differences in both size and edema of the right and left portions of intestine, divided by membranous stricture, were identified from the abdominal wall to the point of the liver dividing laterally from internal segments. The point of stricture of the falciform ligament was visualized clearly as edematous intestines had compressed onto the anterior liver, and this represents an important visual finding in falciform ligament herniation. If a defect in the falciform ligament is caudal to the liver, it will not be readily identifiable. However, in such cases, the diagnosis may still be made by recognizing the constriction of intestine directly under the abdominal wall and directly in the medial axis of the trunk. These specific CT findings, along with a high index of suspicion for this disease entity in cases of unexplained abdominal pain, may aid in diagnosis of herniation through the falciform ligament, enabling more rapid pre-operative diagnosis. Though this diagnosis remains rare, the above clinical presentation and imaging should alert physicians to its possibility.

## Conclusions

We present a rare case of strangulated small bowel due to internal hernia through a congenital defect in the falciform ligament. Our findings on CT imaging assisted with the pre-operative diagnosis and enabled us to rapidly obtain surgical intervention. Early diagnosis may help preclude significant strangulation with unnecessary resection.

## Consent

Written informed consent was obtained from the patient for publication of this case report and any accompanying images. A copy of the written consent is available for review by the Editor-in-Chief of this journal.

## Competing interests

The authors declare that they have no competing interests.

## Authors’ contributions

HS interpreted the data from our patient and was directly involved in the full implementation of this report. SS was jointly involved in the surgical intervention and was a major contributor in writing the manuscript. All listed authors were involved in writing and editing the manuscript, and all authors read and approved the final manuscript.
